# Alien limb syndrome: A Bayesian account of unwanted actions

**DOI:** 10.1016/j.cortex.2020.02.002

**Published:** 2020-06

**Authors:** Noham Wolpe, Frank H. Hezemans, James B. Rowe

**Affiliations:** a Department of Clinical Neurosciences, University of Cambridge, Cambridge, UK; b MRC Cognition and Brain Sciences Unit, University of Cambridge, Cambridge, UK

**Keywords:** Alien limb, Predictive processing, Supplementary motor area, Dual premotor, Action affordance

## Abstract

An alien limb is a debilitating disorder of volitional control. The core feature of alien limb is the performance of simple or complex semi-purposeful movements which the patient reports to be unintentional or unwanted, or occasionally in opposition to their intentions. Theories of the mechanism of alien limb phenomena have emphasised the role of disinhibition in the brain, and exaggerated action ‘affordances’. However, despite advances in cognitive neuroscience research and a large public and media interest, there has been no unifying computational and anatomical account of the cause of alien limb movements. Here, we extend Bayesian brain principles to propose that alien limb is a disorder of ‘predictive processing’ in hierarchical sensorimotor brain networks. Specifically, we suggest that alien limb results from predictions about action outcomes that are afforded unduly high precision. The principal mechanism for this abnormally high precision is an impairment in the relay of input from medial regions, predominantly the supplementary motor area (SMA), which modulate the precision of lateral brain regions encoding the predicted action outcomes. We discuss potential implications of this model for future research and treatment of alien limb.

Alien limb syndrome has attracted a wide public interest due its unusual clinical manifestations (e.g., Mosley, 2011). The term ‘alien limb’ itself implies an enigmatic and dramatic nature of an otherwise debilitating disorder. In the clinical context, alien limb refers to the performance of seemingly purposeful or semi-purposeful movements, which patients report as independent of their own intentions or despite the lack of intention. These movements can sometimes be performed *against* the patient's will, in what has also been termed the ‘anarchic’ limb syndrome (Marchetti & Della Sala, 1998), as infamously but unrealistically depicted in the film Dr Strangelove (1968). The clinical reality is more prosaic, but nonetheless distressing for patients and puzzling for observers.

The central feature for a limb to become alien is the reduced volitional control and diminished sense of agency – that is, the sense that one controls one's own actions (Wolpe & Rowe, 2014). Importantly, the sense of agency is retained by the patient for *other* actions, and is not accompanied by Schneiderian delusions of passivity and external control (Lewis-Smith, Wolpe, Ghosh, & Rowe, 2020). Other clinical and behavioural features can vary significantly between patients. Some patients can be aware of their unwanted action from the outset, whereas others may lack awareness of the action until it is pointed out to them. The response to the unwanted movement can vary from embarrassment or laughter, frustration or surprise, to the denial of ownership of the limb itself (Biran & Chatterjee, 2004). Unwanted movements that retain the sense of limb ownership have been suggested to be phenomenologically and aetiologically different from those that lack ownership (Marchetti & Della Sala, 1998).

There are other clinical disorders with impairment of volitional control that are nonetheless distinct from alien limb (Rowe & Wolpe, 2014). For example, psychogenic movement disorders can include movements that are perceived as involuntary, without an organic neuropathology. They are typically stereotyped movements (Hallett, 2010). Tics are also involuntary movements or vocalisations which are partially independent of patient volition. Tics are stereotypical (Cohen, Leckman, & Bloch, 2013), and can be partially or temporarily suppressed with mental effort or by concentrating on other activities (Cohen et al., 2013). Choreiform movements are a continuous sequence of involuntary, fleeting, migratory muscle contractions (Cardoso, Seppi, Mair, Wenning, & Poewe, 2006). Unlike alien limb, choreiform movements are independent of environmental cues. Xenomelia is a rare syndrome in which patients feel that one or more limbs do not belong to them, sometimes accompanied by a desire for amputation (Brugger, Lenggenhager, & Giummarra, 2013). Importantly, this diminished sense of ownership is not accompanied by involuntary movements and is more closely related to body dysmorphic disorder.

The clinical phenomena of alien limb can arise from diverse neurological disorders (Graff-Radford et al., 2013), including focal lesions such as stroke or a tumour (Biran and Chatterjee, 2004, Doody and Jankovic, 1992, Fisher, 2000, Goldberg and Bloom, 1990). Further, the alien limb phenomena are part of the defining clinical diagnostic criteria for the neurodegenerative corticobasal syndrome, commonly caused by corticobasal degeneration (Alexander et al., 2014, Armstrong et al., 2013).

Neuroimaging and lesion studies have investigated the neural correlates of alien limb. In a case study of the corticobasal syndrome, activity in the inferior frontal gyrus differentiated unwanted alien hand movements from volitional actions of the same hand (Fig. 1A; Schaefer, Heinze, & Galazky, 2010). Both types of hand movements were associated with activity in the motor and premotor cortex, but only ‘alien’ movements evoked brain activity in the inferior frontal gyrus (Schaefer et al., 2010). In a group of patients with the corticobasal syndrome, we found that changes to the pre-supplementary motor area (pre-SMA) and its connections (e.g., prefrontal corpus callosum) were associated with the presence and severity of alien limb (Wolpe et al., 2014). Greater abnormality in the sense of agency (measured in terms of ‘intentional binding’, see Haggard et al., 2002, Wolpe and Rowe, 2014) was similarly related to structural and functional changes in this medial-lateral prefrontal network, centred on the pre-SMA (Fig. 1B; Wolpe et al., 2014). Lesion studies of alien limb have also emphasised the critical role of the medial frontal lobe and anterior corpus callosum in alien limb (Feinberg et al., 1992, Scepkowski and Cronin-Golomb, 2003). However, posterior cortical change may also give rise to alien limb, with lesions in a functional network centred on the precuneus (Darby, Joutsa, Burke, & Fox, 2018).

**Fig. 1:**
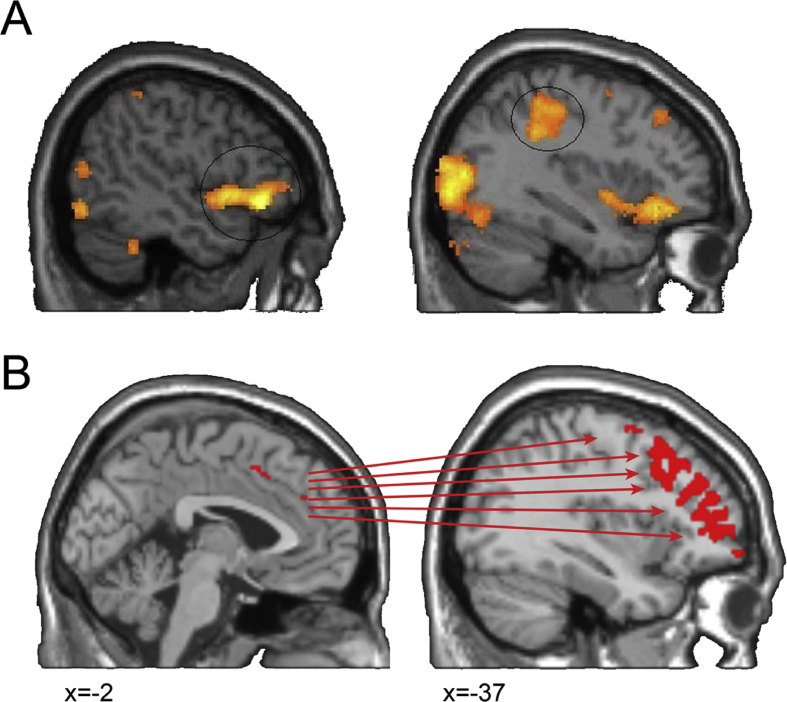
Increased prefrontal activity in alien hand. A) In a patient with the neurodegenerative corticobasal syndrome, unwanted movement of an alien limb was specifically associated with activity in the inferior frontal gyrus (top) and increased activity of the primary motor cortex. Adapted from Schaefer et al. (2010). B) In a group of patients with the neurodegenerative corticobasal syndrome, reduced sense of agency and alien limb severity were associated with structural and functional changes in the pre-SMA and its connectivity with a large prefrontal network. From Wolpe et al. (2014).

Despite a growing body of research in the field of volition and agency in the healthy and clinical populations (Haggard, 2008, Wolpe and Rowe, 2014), the underlying mechanisms of alien limb remain elusive. In this Review, we propose that alien limb can be understood in the context of Bayesian brain theory, and specifically in terms of action plans that have abnormal ‘precision’. We begin with a short review of current theories of alien limb, which we go on to show can be reframed under a unified Bayesian theory of alien limb.

## 1 Current models of alien limb

Motor control theories of alien limb have been influential in recent decades, encompassing aberrant affordances and impairments of response inhibition (Goldberg, 1985, McBride et al., 2013). From this perspective, the alien limb is disinhibited, and even perseverative, with performance of motor schema or ‘stereotypies’ (e.g., Biran, Giovannetti, Buxbaum, & Chatterjee, 2006). When these motor phenomena are accompanied by intact action monitoring, the experience of ‘alienness’ occurs (Biran et al., 2006).

**Fig. 2:**
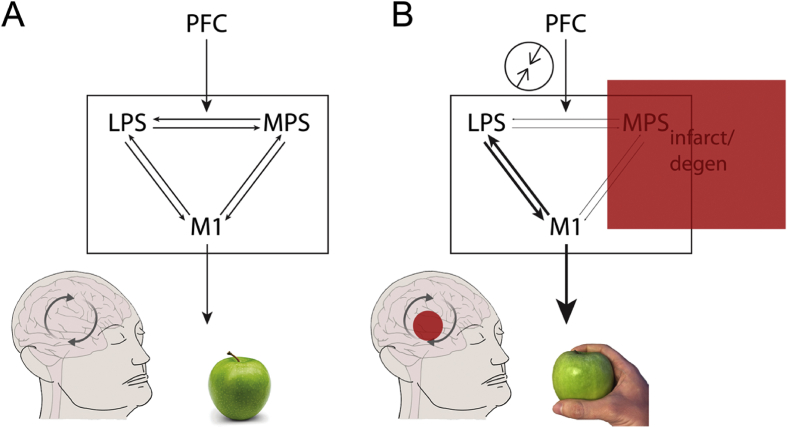
Alien limb as an imbalance between the two premotor systems, after Goldberg and Bloom (1990). A) A voluntary action typically relies on the interaction between the lateral premotor system (LPS), including the premotor and parietal cortices, and medial premotor system (MPS), which includes the supplementary motor area. While the LPS is responsive, the MPS is anticipatory in nature. Their interaction is modulated by higher cortical regions within the prefrontal cortex (PFC), which takes into account contextual signals. The interaction eventually determines the type of movements to be carried out by the primary motor cortex (M1), such as whether to grasp an apple an eat it, depending for example on the appropriateness and need for a certain action. B) In alien limb, lesion (e.g., infarct or degeneration) to the MPS leads to a loss of balance, and the performance of motor stereotypies, such as grasping an apple, even when these are not appropriate. The experience of ‘alienness’ develops as a result of the discrepancy between action monitoring systems in the PFC and LPS signals.

There are contrasting accounts of the origins of the disinhibition in alien limb. An influential theory of voluntary action proposes a continuous interaction between two functionally distinct premotor systems (Goldberg, 1985). The lateral premotor system, centred on the premotor cortex, provides a responsive mode to produce actions driven by explicit external inputs. It associates external stimuli, such as objects, with contextually relevant actions. For example, the visual signals of an apple on a table automatically generate a motor plan to reach and grasp the apple. By contrast, the medial premotor system, centred on the supplementary motor area (SMA), provides a generative mode to produce actions from intrinsic motivational signals (Goldberg, 1985). Normal behaviour relies on a balanced interaction between these two systems, controlled by higher level decision-making regions e.g., the prefrontal cortex (Fig. 2A). The execution of a lateral premotor motor plan is partially dependent on motivational signals (am I hungry?) or context, such as whether it is appropriate to make a particular action (e.g., is it someone else's apple?). One can also operate in an ‘automatic’ responsive mode (grasp the apple and eat it) or act independently from old stimulus–response associations (placing the apple at the palm of one's hand). For an alien limb, damage to the medial premotor system leads to a disinhibition of the lateral premotor system and hence the inappropriate production of ‘stereotypical’ actions (Goldberg & Bloom, 1990), such as grasping an apple (Fig. 2B).

The dual premotor theory was largely based on lesion studies in humans and neurophysiological data in animal models. It has strongly influenced the neuroscience of volition. However, theoretical and experimental studies have challenged the proposed dichotomy of the motor systems. For example, the role of the SMA of the medial frontal system in ‘pure’ self-generated movements has been challenged (discussed in Passingham, Bengtsson, & Lau, 2010), as the SMA has been shown to be critical for generating environmentally driven action sequences (Nachev, Kennard, & Husain, 2008). Moreover, neuroimaging methods have emphasised the importance of network connectivity and integration, rather than a set of localised functions (Bassett and Bullmore, 2009, Rowe and Siebner, 2012).

**Fig. 3:**
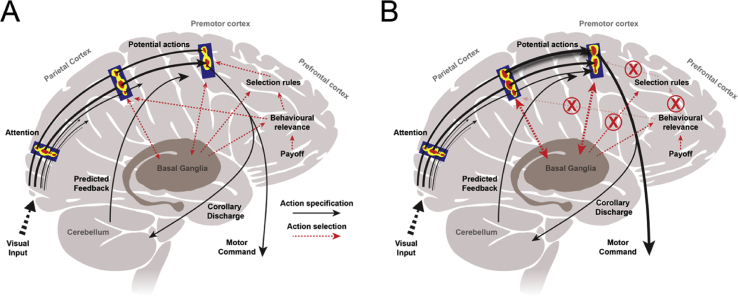
Alien limb as exaggerated affordance, after Cisek (2007) and Cisek and Kalaska (2010). A) Voluntary action results from ‘affordance competition’. Visual input is processed in the occipital cortex, and immediately triggers the competition between different potential actions (‘affordances’), which are increasingly specified in terms of their spatiotemporal features in a lateral parietal-premotor network (black arrows). The behaviourally most relevant and appropriate action is selected by biasing the competition through basal ganglia and prefrontal input. B) In alien limb, damage to frontal regions impairs the brain's ability to select (or inhibit) a behaviourally relevant action, leading to an exaggerated affordance (McBride et al., 2013).

An alternative theory emphasises the role of fronto-parietal networks in selecting and specifying *potential* voluntary actions (Cisek, 2007, Cisek and Kalaska, 2010). Sensory, cognitive and motor processes are proposed to be processed in parallel rather than in serial as formerly considered. On this account, external sensory stimuli activate the representations of potential actions – termed ‘affordances’ (Gibson, 1979). These are processed in parallel and compete against each other for selection, while additional sensory information is acquired (Fig. 3A). An action is eventually selected by biasing the competition towards one particular action through the influence of decision-making systems, including prefrontal and striatal circuits (Cisek, 2007, Cisek and Kalaska, 2010). An alien limb would arise from ‘exaggerated affordance’, or inadequately inhibited affordances (McBride et al., 2013). This may result from damage to the connections of prefrontal regions, leading to improper biasing of the competition and performance of behaviourally irrelevant and even inappropriate actions (Fig. 3B).

The proposed abnormality in affordance competition in alien limb has provided a set of testable hypotheses. It is consistent with neurophysiological data and a broader conceptual framework for behavioural decisions in terms of accumulation-to-threshold models (Gold & Shadlen, 2007). However, as the theory emphasises the failure of frontal regions to successfully bias the affordance competition, it cannot easily account for alien limb cases that result from more posterior lesions. The SMA has been proposed to be central for exaggerated affordance (McBride et al., 2013), however, its exact role within the fronto-parietal network for affordance competition remains elusive (Cisek & Kalaska, 2010).

The dual premotor theory and action affordance model have been influential for the neuroscience of voluntary action (Haggard, 2008). However, they are limited in terms of computational, algorithmic or implementational levels (Marr, 2010) and in generalisation to other normative aspects of behavioural control. For example, the dual premotor theory emphasises an implementational level in terms of regional neuroanatomy, but is anatomically restricted and overlooks the interconnected, distributed or connectionist nature of brain function. By contrast, the affordance competition model provides an explicit (although partial) implementational and algorithmic account, but remains unclear at the computational level, i.e., does not directly answer what normative problem the system attempts to solve.

An algorithmic framework that integrates the normative computational and implementational levels could help better understand the alien limb phenomenon. One such framework is offered by the ‘comparator’ model of motor control and the sense of agency. The model posits that the motor system aims to minimise the difference between a ‘desired state’ and the body's current state (Blakemore et al., 2002, Frith, 1987). Inverse models resolve this discrepancy (error term) by generating an appropriate motor command (Wolpert, Diedrichsen, & Flanagan, 2011). An efference copy of the motor command is used to generate a prediction by forward models about the sensory consequences of movement. The prediction is in turn used to quickly update and correct movements to achieve their goal and minimise the difference between the desired and current state. The predicted sensory consequence is continuously compared to the actual sensory input, enabling the system to discriminate between internally and externally generated sensory stimuli. This comparison is proposed to underlie the sense of agency, as a large discrepancy between the predicted and actual sensory input indicates that the sensation was externally generated, independent of one's own volition. Within this framework, alien limb has been suggested to arise from motor commands that are no longer coupled with the patient desires or intentions (Blakemore et al., 2002). It is possible that impairments in generating efference copies might also underlie alien limb. Biran et al. (2006) suggested that alien movements lack a desired state and are not accompanied with a predicted state, because of a disconnection between the forward models and sensory feedback, which hampers the comparison of prediction and sensory feedback. The combination of this disconnection and the patient remaining aware of the changing state would lead to an alien limb (Biran et al., 2006).

Although the comparator model can describe a failure of motor processing in alien limb, a simple comparator fails to capture important aspects of the sense of agency and its role in alien limb. For example, people can attribute actions to themselves despite a mismatch between the predicted (efference copy derived-) and actual outcome (see Synofzik, Vosgerau, & Newen, 2008). A weakness of the comparator model of alien limb is the omission of post-dictive accounts of agency. For example, a match between intentions and beliefs about sensory causes with sensory effects can itself at times be sufficient for self-attribution of action (Wegner, 2003). In other words, there are cases in which the comparator is not required for sense of agency to arise. By focusing on the comparison between predicted and actual outcome, the comparator model fails to capture the complex post-dictive, contextual aspects of a voluntary action and the sense of agency (Wegner, 2003). These findings have led to an extended comparator model of agency, with comparisons of different levels of internally and externally generated cues (Synofzik et al., 2008).

An alternative theory which captures the multi-level comparisons of sensory and predicted information is based on the ‘predictive processing’ theory of perception and behaviour (Friston, FitzGerald, Rigoli, Schwartenbeck, & Pezzulo, 2017). We propose that predictive processing offers a new theoretical framework for a unified account of alien limb at the computational, algorithmic and implementation levels (Marr, 2010). We argue that core elements of the earlier models can be reframed in terms of hierarchical predictive processing of the sensory consequences of action.

We set out how disinhibition of motor plans in alien limb can be operationalised under a unifying Bayesian brain hypothesis embedded in predictive processing. We first describe the basic underlying principles of voluntary movement as a process of ‘active inference’ (Adams, Shipp, & Friston, 2013), and then put forward our main proposition that prior beliefs about a specific action, encoded in a hierarchical parietal-premotor network, are afforded unduly high precision due to a disconnection from modulatory medial cortical regions.

## 2 A Bayesian brain theory of motor systems

Advances in computational and cognitive neuroscience have highlighted the role of hierarchical prediction and inference in brain function, across many areas of perception, movement and cognition (Pouget et al., 2013, Seth, 2015, Tenenbaum et al., 2011). The origins of predictive processing lie in James' (1890) ideomotor theory of action control, and von Helmholtz's (1866) view of the brain as a statistical inference engine whose function is to infer the probable causes of sensory input. These early theories emphasise that action and perception are both governed by top-down predictions. These predictions were later tied to prior beliefs,^1^ which are combined with sensory information to infer the mostly likely causes of sensory input (see also Hommel et al., 2001, Prinz, 1997).

The fundamental concept of Bayesian brain theories is that the brain seeks to maximise the evidence for a ‘generative model’ of its environment, such that prior beliefs about states of the environment are consistent with the observed sensory evidence (Friston, 2010, Friston and Kiebel, 2009). An important motivation for pursuing such a Bayesian framework is its ability to account for a wide range of both normative and apparently suboptimal behaviour. According to the ‘good regulator theorem’ (Conant & Ashby, 1970), the brain can only regulate its environment effectively if it establishes a good model of that environment. This implies that if the brain maintains aberrant prior beliefs, it will fail to effectively regulate its environment, which manifests as false inferences and maladaptive behaviour.

Neuropsychological deficits have indeed been characterised as Bayes-optimal inference with abnormal prior beliefs (Parr et al., 2018, Schwartenbeck et al., 2015). As we argue below, a specific set of suboptimal priors may give rise to alien limb. The key questions that we seek to answer are thus as follows. First, what type of prior beliefs would lead to alien limb phenomena? Second, what are the biological substrates of these priors, and how can we relate these substrates to previous clinical and experimental findings?

Before we address these questions, we elaborate on how the brain might optimise its generative model of the environment, and how this process encompasses action. According to predictive coding, this optimisation is achieved by minimising the ‘prediction error’ signal – that is, the difference between top-down predictions and bottom-up sensory evidence (Spratling, 2017). Brain regions are organised in hierarchies, and each level of the hierarchy consists of a population of ‘error units’ and ‘prediction units’. Error units receive top-down signals from prediction units in the level above, and compare this prediction to the sensory belief of the current level (Friston, 2008). The ensuing prediction error is then signalled up to the higher level to update the prediction unit. Prediction errors can be minimised by updating the belief so as to better fit the sensory inputs (perceptual inference).

Prediction errors can also be minimised actively, by performing actions to change the sensory input itself. This is known as ‘active inference’. Note that on active inference – in contrast to the comparator model – there are no functionalist ‘goals’ or ‘motor commands’; the brain is solely concerned with minimising prediction errors across hierarchical levels of abstraction, either through perception or action (Adams et al., 2013). This does not mean that higher order consequences of action, such as emotional states (Seth, 2015), are irrelevant, but merely that they are framed within the context of prediction error minimisation.

For illustration, consider a hierarchy of three levels. The upper level of the hierarchy represents temporally-extended, domain-general, multi-modal beliefs. These cannot be easily conceived as specific predictions for specific sensations, as they are abstract and include high-level self-referential information (e.g., that it is my birthday today). These high-level predictions are relayed hierarchically to lower levels that are more modality-specific and that increasingly relate to predicted sensations: first to intermediate temporal extent and contextual specificity (e.g., that I am eating a cake), and then to immediate proprioceptive predictions (e.g., that I am raising my spoon). The proprioceptive predictions propagate down from the upper motor neuron in the motor cortex through the corticospinal tract and to the ventral horn of the spinal cord, where they are compared with proprioceptive input (Adams et al., 2013). In the lower motor neuron, the difference between proprioceptive prediction from the upper motor neuron and proprioceptive signal from the sensory neuron causes its activation and the initiation of a movement (Friston et al., 2012). In the following section, we discuss how specific actions are carried out or inhibited; a process which is crucial for understanding of alien limb.

## 3 Precision modulation determines action selection

To understand how an action is carried out or inhibited, consider that both prior beliefs and prediction errors are encoded as a probability distribution, rather than discrete events. The belief at each level of the hierarchy is equated to a Bayesian posterior distribution, computed from the previous integration of top-down predictions with bottom-up prediction errors. This integration follows a Bayes optimal integration, in which less precise (or more uncertain) signals are weighted less than high precision signals. Precision is proposed to be a biologically relevant scale, encoded by synaptic gain (inverse of the variance in fluctuation of neuronal activity, see Friston et al., 2012). Relatively precise priors are therefore more likely to ‘overwhelm’ sensory input and be fulfilled through action. Thus, on active inference, action selection is achieved by modulating the relative precision of prior beliefs and sensory inputs (Pezzulo, Rigoli, & Friston, 2018).

On active inference, object affordance competition is reframed as the association of specific bottom-up prediction errors (sensory evidence) with high precision of specific predictions of outcomes. As in the example above, sensory evidence for an apple may induce different predictions about action outcomes encoded in hierarchical premotor-parietal circuits. However, the highest precision is eventually afforded to a higher-level belief that it is time to eat, and lower-levels beliefs about predicted sensations from an ensuing grasping movement. On this interpretation, action affordance competition becomes a competition for the highest precision of intermediate level beliefs specifying predicted action outcomes, e.g., from touching, grasping or punching an apple.

Two main mechanisms modulate the precision of these intermediate level beliefs: top-down modulation of high-level beliefs and modulation from lateral regions. First, intermediate level beliefs about action outcomes are contextualised by high-level priors about semantic and episodic context. Higher hierarchical levels operate at longer timescales and enable goals and long-term strategies for an exchange with the world to be fulfilled (Kiebel et al., 2008, Murray et al., 2014). This means that higher hierarchical levels provide top-down prior constraints on transitions at the intermediate sensorimotor level, so that actions fulfil (and remain consistent with) high-level prior beliefs about outcomes (Pezzulo et al., 2018). This high-low gradient is thought to be reflected mainly in the anterior-posterior cortical axis (Friston, 2010). For example, intermediate level beliefs about action outcomes in the premotor cortex may be modulated by their contextual relevance and appropriateness in dorsolateral prefrontal regions. If it is after lunch, an individual remembers she has purchased an apple, and others around her are eating an apple, this would generally cause an increase to the precision of high-level beliefs that she is also eating an apple, thereby increasing the intermediate-level beliefs about reaching for the bag to grasp the apple. This action would eventually be fulfilled, unless other more precise high-level beliefs are opposed to this prediction, e.g., that the individual is full.

**Fig. 4:**
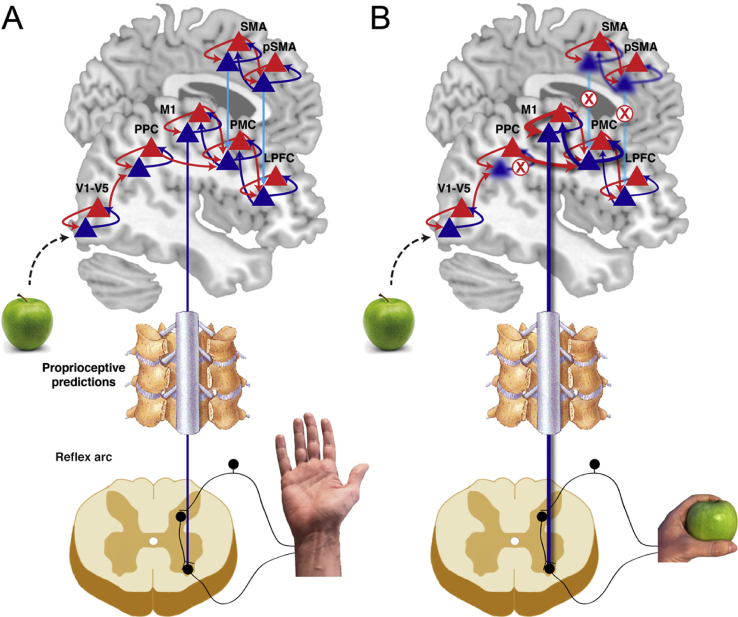
Predictive processing account of alien limb. A) Normal behaviour relies on hierarchical brain networks encoding predictions (blue) and prediction errors (red). Prediction units are ensembles of neurons whose activity encodes the probability distribution of anticipated action outcomes. The predicted outcome is encoded in increasing level of complexity and abstraction in the posterior-anterior brain axis. At each level of the hierarchy, predictions from a higher level are compared with predictions from the current level by the error units. Prediction errors are forwarded up the hierarchy, while predictions propagate down the hierarchy. In movement, prediction errors are minimised by changing bottom-up sensory evidence. Specifically, predictions that are afforded sufficient precision propagate down the hierarchy where they induce movements by the motor neurons through a mechanism similar to the classical reflex arc, where predictions about proprioceptive signals are compared with actual proprioceptive input. B) We propose that networks in medial brain regions encode predictions of the precision of predictions in lateral regions (light blue). The principal mechanism in alien limb is then an impairment in the lateral connections between medial and lateral regions, either because of white matter deficits or damage to grey matter in medial frontal regions (we do not formally distinguish between damage to grey matter or white matter). Similarly, damage to lateral regions such as posterior parietal cortex, can afford certain beliefs about action outcomes (i.e., affordances) with high precision. This leads to unmoderated, abnormally high precision of certain outcomes that are not consistent with motivational and/or contextual relevance.

Second, lateral connections play a key role in tuning the precision of beliefs, possibly by changing the synaptic gain (Friston, 2008). Accordingly, the medial-lateral axis may interact for optimising the precision of these beliefs, e.g., based on motivational signals (see Duverne & Koechlin, 2017; Kouneiher, Charron, & Koechlin, 2009). Considered together with the dual premotor hypothesis, we propose that the medial premotor system operates so as to modulate the precision of predictions in the lateral premotor system (Fig. 4A). The highly-connected medial and lateral networks thus encode related but biologically distinct quantities: the lateral networks encode hierarchical predictions about sensory states, whereas the medial networks encode the predicted precision of these lateral predictions, ordered hierarchically equivalent to the lateral network. For example, the (medial) SMA influences the intermediate-level predictions or affordances encoded in the (lateral) premotor cortex by modulating its precision. Their connections can be modulatory in nature, e.g., mediated by synapses between en passant boutons of precision prediction units of the medial system and post-synaptic receptors of prediction units of the lateral system (Friston et al., 2012). Ultimately, the precision of these predictions in the lateral premotor region will determine whether and which action will be carried out via its connections with the motor cortex.

This active inference model sheds new light on previous experimental findings of the experience of volition from direct cortical stimulation. Classical stimulation studies in humans showed that SMA stimulation evokes involuntary contralateral movements, such as grasping and groping (Penfield & Keasley, 1951). This finding has been confirmed (Fried et al., 1991) and it was further shown that low-intensity stimulation of the SMA can sometimes lead to human participants reporting an ‘urge’ or intention to move a specific body part (Fried et al., 1991; but see Trevisi et al., 2018). In some cases, stimulation led patients to believe they had moved a specific body part (Fried et al., 1991). Interestingly, similar but less specific responses of intentionality were found in a study with nine patients with direct stimulation of (lateral) posterior parietal cortex (Desmurget et al., 2009). Parietal stimulation even at higher intensities, did not evoke actual movements, despite the patients believing a movement has occurred (Desmurget et al., 2009). We interpret this stimulation of the posterior parietal cortex as ‘artificially’ increasing the precision of low and then intermediate level beliefs about action outcomes (i.e., affordance), resulting in the experience of an intention to perform the specific action to fulfil this belief, e.g., “I felt the desire to lick my lips” (Desmurget et al., 2009). Patients believed a movement had occurred because stimulation evoked a lateral fronto-parietal activity similar to the physiological hierarchical belief propagation. However, increasing the stimulation intensity did not induce an action because the precision of this affordance is still subject to the modulation of motor precision by medial regions, such as the SMA. The SMA reduces this artificially high precision, in line with current motivational cues, and through its connections with the adjacent pre-SMA that acts as a ‘brake’ to prevent inappropriate actions (Nachev et al., 2007, Sumner et al., 2007). High intensity stimulation of the SMA, on the other hand, could cause an action (Fried et al., 1991), by inappropriately increasing the precision of a specific affordance (intermediate-level prediction) through modulatory signals. Since the precision modulation is largely determined by adjacent medial regions, high intensity stimulation of the SMA, similar to direct stimulation to the lateral premotor cortex (Desmurget et al., 2009), would lead to an action.

## 4 Alien limb from abnormal motor precision

The predictive processing framework for action leads to specific predictions about the mechanisms of alien limb phenomena. Normal behaviour requires higher-level beliefs about intended outcomes to be held with sufficient precision, so as to be propagated down a cortical hierarchy and continuously ‘explain away’ prediction errors from lower sensorimotor levels. In the three-level hierarchy described above, when intermediate sensorimotor predictions are afforded a pathologically high precision, they will be maintained despite contradictory predictions from higher levels. When intermediate sensorimotor priors are overly precise, they fail to be informed by higher-level goals and beliefs. Instead, they may evolve predominantly from the temporally confined somatosensory input from lower hierarchical levels, including salient perceptual cues that gave rise to the concept of ‘affordances’.

Under active inference, exaggerated affordance would arise where low-level prediction units have abnormally high precision, propagating up the cortical hierarchy without being explained away by higher level beliefs, and therefore a movement is induced. The movement that is induced is inconsistent with the patient's contextually determined intentions and goals, represented by high level beliefs. We propose that this inconsistency leads to the subjective experience of alienness (see also Edwards, Adams, Brown, Pareés, & Friston, 2012). The model predicts erratic and disorganised stimulus-dependent behaviours in this situation, such as grasping and other ‘utilisation’ behaviour that are common in alien hand syndrome.

What might lead to the abnormally high precision of intermediate-level sensorimotor predictions? In outlining the principles of the dual premotor and affordance competition theories, we described two types of lesion effect that are to be re-interpreted under active inference. First, medial damage would impair the modulation of affordance precision competition in the lateral system (Fig. 4B, medial view). Alien limb is commonly associated with medial frontal lesions, such as in the SMA (Scepkowski & Cronin-Golomb, 2003), which would impair its modulation over the lateral premotor cortex, and consequently afford the leading affordance sufficient precision to propagate through the motor cortex down the corticospinal tract and induce a movement. Lesions to the pre-SMA or its connections (Wolpe et al., 2014) impair the precision modulation of prefrontal regions, which would in turn be unable to influence the intermediate-level prediction units (cf. bias affordance competition, as in Cisek, 2007). Such a damage to medial frontal regions or their lateral connections has indeed been associated with alien limb severity in the neurodegenerative corticobasal syndrome (Wolpe et al., 2014). The abnormally increased functional connectivity between these medial frontal regions and lateral frontal and prefrontal areas may reflect their inefficient communication (Wolpe et al., 2014). The posterior medial region of precuneus is also relevant, and forms a central hub for a posterior brain network impaired by lesions that cause an alien limb (Darby et al., 2018).

Second, direct damage to lateral regions that encode predictions about action outcome lead to their abnormal precision, by making them less responsive to modulatory signals. For example, damage to lateral regions in the parietal cortex can lead to purposeless arm movement with wandering and levitation (Leiguarda, Starkstein, Nogues, Berthier, & Arbelaiz, 1993). Such a damage enables relatively low-level predictions or beliefs to propagate up the hierarchy through prediction errors with exaggerated precision (Fig. 4B, lateral view). Interestingly, more posterior lesions have been proposed to be phenomenologically distinct from frontal alien limb, in that posterior alien limb cases may lack the degree of purposefulness of the actions seen in frontal cases (Scepkowski & Cronin-Golomb, 2003).

The apparent purposefulness or complexity of alien movements may be determined by the localisation of the lesion on the anterior-posterior axis within the hierarchical predictive networks. Specifically, as more anterior regions typically represent higher levels in the predictive processing hierarchy, anterior lesions may lead to more complex, quasi-purposeful movements. By contrast, more posterior lesions may lead to primitive stereotypical movements. Damage to the SMA or its connections for example, which is commonly associated with alien limb (Scepkowski & Cronin-Golomb, 2003), would lead to the dominance of intermediate level predictions. The dominance of more immediate predictions over temporally extended predictions at higher levels might explain why alien limb phenomena are usually short, isolated, simple movements, rather than long dissociative states of extended behaviours (Lewis-Smith, Wolpe, Ghosh, & Rowe, 2020).

Despite the examples described above, one must also consider that brain lesions from stroke or neurodegeneration only rarely lead to alien limb. One factor is the exact localisation of the lesion. Degeneration of rostral prefrontal cortex in frontotemporal dementia is not associated with alien limb, despite other behavioural changes such as risky decision-making and disinhibition (Lansdall et al., 2017, Passamonti et al., 2018). We propose that for alienness to develop, there must be a discrepancy between *intact* upper levels of the hierarchy (anatomically anterior/rostral) and imprecise intermediate levels (anatomically premotor/SMA). A corollary is that patients are aware of the discrepancy between their intentions and actual movement (see Biran et al., 2006). In contrast to frontotemporal dementia, corticobasal degeneration leads to more severe degeneration of intermediate (premotor/SMA) levels, with relative preservation of rostral prefrontal cortex (see below).

There are indeed strong associations between alien limb and medial prefrontal cortex, however, no specific lesion location has been shown to be ‘necessary’ for alien limb (Scepkowski & Cronin-Golomb, 2003). Plasticity-related changes after the lesion occurs (whether neurodegenerative or ischaemic) may determine whether alien limb develop or not. Further, even for a given lesion in a region like the SMA, the specific layer(s) that are damaged and their white matter connections may also influence the development of alien limb. Superficial and deep cortical layers, for example, have different roles in the transmission of feedforward and feedback information underlying prediction and prediction error, respectively (Friston and Kiebel, 2009, Zeki and Shipp, 1988).

White matter damage is also associated with alien limb. Callosal damage is particularly common in patients with alien limb (Scepkowski and Cronin-Golomb, 2003, Wolpe et al., 2014), with or without concurrent grey matter damage. White matter damage leading to a functional disconnection may contribute to alien limb through the failure to relay hierarchical prediction and prediction errors. However, it has been suggested that ‘pure’ callosal alien limb is phenomenologically different from frontal alien limb, with intermanual conflict and interference from the non-dominant alien hand rather than semi-purposeful environmentally-dependent behaviours (Feinberg et al., 1992). Others have suggested that pure callosal alien limb requires a disconnection between the bilateral SMA and motor areas leading to a similar phenomenology (Geschwind et al., 1995, reviewed in; Scepkowski & Cronin-Golomb, 2003).

We have so far focussed on structural lesions and abnormal connections of hierarchical brain networks as determinants of alien limb, but both neurophysiological impairments and neurotransmitter deficits could affect the precision of intermediate priors. A central determinant of the precision of prior beliefs is neuromodulation, where signal gain is regulated at the level of the synapses (Edwards et al., 2012, Friston et al., 2012). In the corticobasal syndrome in which alien limb is a common diagnostic feature, several relevant neurotransmitter deficits occur (Murley & Rowe, 2018). There is loss of dopaminergic neurons in the substantia nigra and striatum, and mesocortical pathway (Oyanagi et al., 2001, Pirker et al., 2015). Dopamine influences the precision of prior beliefs for action, both empirically (Schwartenbeck et al., 2015, Wolpe et al., 2015) and in theoretical models of predictive processing (Friston et al., 2012). However, changes in cortical GABA and glutamate in corticobasal syndromes may be more directly relevant. GABA and glutamate moderate the gain function in superficial pyramidal cells in response to feedback, and their interactions with deep pyramidal cells within each cortical column (Bastos et al., 2012). The changing balance of GABA/glutamate will therefore affect the relative precision of feedforward versus feedback information. The site and relative loss of these neurotransmitters in corticobasal syndromes differ from other common forms of frontotemporal lobar degeneration and Parkinson's disease (Murley & Rowe, 2018), enabling the specificity of alien limb in corticobasal syndromes whether arising from corticobasal degeneration pathology or mimics from atypical patterns of Alzheimer's disease (Alexander et al., 2014).

## 5 Comparison to other disorders of volition

There are other neuropsychiatric disorders which impair voluntary control with overlapping neuroanatomical lesions, but without alien limb. Behavioural variant frontotemporal dementia, for example, causes impulsivity, stereotypy and perseveration, utilisation and grasping behaviours (Rowe & Wolpe, 2014). Such behaviours span a wide variety of complexity, from stereotypies and environmentally bound behaviours to higher-order analogues like clock-watching, obsessional or compulsive actions, and loss of decorum (Nyatsanza et al., 2003). However, patients do not experience these behaviours as ‘alien’, we argue, because the damage is to higher levels in the cortical hierarchy, with reduced top-down influences from high-level beliefs onto intermediate priors (Hughes, Ghosh, & Rowe, 2013).

Tourette syndrome causes involuntary ‘tics’, which can only partially be voluntarily suppressed. The Bayesian account of Tourette syndrome (Rae, Critchley, & Seth, 2019) identifies abnormally precise priors for action at an intermediate level of the hierarchy, leading to movement that was not predicted by high-level brain regions. However, the model of alien limb is tied to different neuroanatomical networks, and has dissociable behavioural implications. Tourette syndrome is associated with increased excitatory activity from the SMA to the putamen, and reduced striatal inhibition, which lead to relatively precise priors for action in the putamen. This leads to the expression of repetitive and recurrent patterns of behaviour represented in the putamen (the tics). Although the severity of tics can vary as a function of environmental, social and psychological factors, the nature of the movement itself is rarely influenced by environmental cues such as object affordances. This contrasts with alien limb in which the abnormal precision leads to erratic behaviour that is driven by salient sensory inputs.

A third condition with unwanted actions and that has been described in terms of overly precise priors is psychogenic movement disorders. There are some similarities to the proposed mechanism of abnormal motor symptoms in psychogenic movement disorders (such as tremor or dystonia) and our account for alien limb, for example, the abnormally high precision of intermediate-level priors in premotor cortex (Edwards et al., 2012). The key difference, however, is that motor symptoms in psychogenic movement disorder have been attributed to a misallocation of attention at the level of specific intermediate-level priors. This is proposed to be mediated by increased synaptic gain as a result of abnormal modulation from the dorsolateral prefrontal cortex (Edwards et al., 2012).

## 6 Future considerations

The attempt to explain volitional disorders in terms of predictive processing and active inference (with abnormal hierarchical beliefs, prediction, and inference) is ambitious in its aim to provide a unified account of brain structure and function. Despite a growing number of studies providing supportive evidence for the theory, psychophysical and physiological validation is required. To test the hypothesis, future studies will benefit from the combination of behavioural, neuroimaging and computational methods in both healthy population and in patients. For example, we hypothesise that experimental lesions in the connections between SMA and lateral premotor cortex; or electrical stimulation of the premotor cortex would promote alien limb movements (c.f. Desmurget et al., 2009). It is uncertain whether the involuntary movements triggered by TMS to the primary motor cortex are phenomenologically similar to alien limb. TMS-induced movements are normally simple flexion/extension movement that lack purposefulness. It remains an open question whether TMS can induce more complex, semi-purposeful movements, for example through high intensity stimulation of the premotor cortex. The volitional aspect makes it not practical to study these questions in non-human subjects. However, there is scope for better accounts of the phenomenology of alien limb in humans, the degree to which they can be induced, and their association with localised brain abnormalities. Specific predictions of the model may be testable in cross-sectional observational studies, for example that longer more complex alien limb movements are associated with more rostral frontal brain lesions than simple actions.

A challenge for the future is to translate these hypotheses into effective therapies for patients affected by an alien limb. The nature of the common lesions that lead to alien limb make conventional therapeutic options seem unlikely. Pharmacological modulation of contributory neurotransmitter deficits may provide temporary and partial alleviation, but remains to be tested in this patient group.

In summary, we have proposed a novel mechanistic account for alien limb, drawn from predictive processing theory for brain structure and function. Specifically, we suggest that alien limb principally results from a change in the hierarchically organised lateral and medial brain networks. The medial network establishes the precision of predictions, but in alien limb it is unable to properly modulate the precision of predictions of action outcome encoded in a lateral network, including the lateral parietal cortex and premotor cortex. Lesions of the SMA lead to unduly high precision of intermediate level predictions of action outcome, which in turn propagate down to the motor cortex to induce semi-purposeful movements. These movements are perceived as involuntary, as they are ‘at odds’ with high level contextual beliefs that are encoded in the rostral and dorsolateral prefrontal cortex. This account of alien limb raises testable hypotheses for future research, which may pave the way for a better understanding of human volition and its impairment and ultimately for therapeutic avenues that may be offered to patients with alien limb.

## CRediT authorship contribution statement

**Noham Wolpe:** Conceptualization, Visualization, Writing - original draft, Writing - review & editing.

**Frank H. Hezemans:** Writing - original draft, Writing - review & editing.

**James B. Rowe:** Funding acquisition, Supervision, Writing - original draft, Writing - review & editing.

## Footnotes

1 We use the term ‘belief’ in the technical sense of Bayesian inference, which is not necessarily the same as the colloquial meaning of belief as a consciously held propositional attitude.
